# Predictive Factors of Plasma HIV Suppression during Pregnancy: A Prospective Cohort Study in Benin

**DOI:** 10.1371/journal.pone.0059446

**Published:** 2013-03-15

**Authors:** Lise Denoeud-Ndam, Camille Fourcade, Aurore Ogouyemi-Hounto, Angèle Azon-Kouanou, Marcelline d'Almeida, Alain Azondékon, Marouf J. Alao, Véronique Dossou-Gbété, Aldric Afangnihoun, Pierre-Marie Girard, Michel Cot, Djimon-Marcel Zannou

**Affiliations:** 1 UMR 216, Institut de Recherche pour le Développement and Université Paris Descartes, Paris, France; 2 Laboratoire National de Référence, Programme National de Lutte contre le Sida, Cotonou, Benin; 3 Centre de Traitement Ambulatoire, Centre National Hospitalier Universitaire Hubert Koutoukou Maga, Cotonou, Benin; 4 Service de Pédiatrie, Centre National Hospitalier Universitaire Hubert Koutoukou Maga, Cotonou, Benin; 5 Service de Pédiatrie, Hôpital d'Instructions des Armées, Cotonou, Benin; 6 Service de Pédiatrie, Hôpital de la Mère et de l'Enfant Lagune, Cotonou, Benin; 7 Clinique Louis Pasteur, Porto-Novo, Benin; 8 Centre de Traitement Ambulatoire, Hôpital de zone de Suru Léré, Cotonou, Benin; 9 Service des Maladies Infectieuses et Tropicales, Hôpital Saint-Antoine, Paris, France; 10 Université Pierre et Marie Curie, Paris, France; 11 Faculté des Sciences de la Santé, Université d'Abomey-Calavi, Cotonou, Benin; Alberta Provincial Laboratory for Public Health/University of Alberta, Canada

## Abstract

**Objective:**

To investigate the factors associated with HIV_1_ RNA plasma viral load (pVL) below 40 copies/mL at the third trimester of pregnancy, as part of prevention of mother-to-child transmission (PMTCT) in Benin.

**Design:**

Sub study of the PACOME clinical trial of malaria prophylaxis in HIV-infected pregnant women, conducted before and after the implementation of the WHO 2009 revised guidelines for PMTCT.

**Methods:**

HIV-infected women were enrolled in the second trimester of pregnancy. Socio-economic characteristics, HIV history, clinical and biological characteristics were recorded. Malaria prevention and PMTCT involving antiretroviral therapy (ART) for mothers and infants were provided. Logistic regression helped identifying factors associated with virologic suppression at the end of pregnancy.

**Results:**

Overall 217 third trimester pVLs were available, and 71% showed undetectability. Virologic suppression was more frequent in women enrolled after the change in PMTCT recommendations, advising to start ART at 14 weeks instead of 28 weeks of pregnancy. In multivariate analysis, Fon ethnic group (the predominant ethnic group in the study area), regular job, first and second pregnancy, higher baseline pVL and impaired adherence to ART were negative factors whereas higher weight, higher antenatal care attendance and longer ART duration were favorable factors to achieve virologic suppression.

**Conclusions:**

This study provides more evidence that ART has to be initiated before the last trimester of pregnancy to achieve an undetectable pVL before delivery. In Benin, new recommendations supporting early initiation were well implemented and, together with a high antenatal care attendance, led to high rate of virologic control.

## Introduction

Despite the fact that in Sub-Saharan Africa, mother-to-child transmission (MTCT) of HIV has substantially decreased over the last decade, it continues to contribute to the disease's burden in many countries. It has been estimated that approximately 300 000 infants have been infected in Africa in 2011 [1] and more than half of them were expected to die before the age of two [2]. The natural MTCT rate without any intervention has been estimated between 30% and 45% depending on breastfeeding [3], and such transmission can occur at various periods including pregnancy (9%), delivery (16%) and post-partum, via breastfeeding (5%) [4]. When adequate prevention strategies are undertaken, MTCT rates can be reduced to less than 2% [5], [6], [7]. One of the main objectives for HIV prevention and control as pointed out by the UNAIDS is the “virtual elimination of MTCT”, which is planned to be achieved by 2015 [1]. The elimination of MTCT involves several measures, updated by the World Health Organization in 2010 [8]. The key element is that all HIV-infected women should receive an antiretroviral therapy (ART), started at least from 14 weeks of gestation, and continued until the end of the breastfeeding period. The infant should also benefit from ART.

Prevention strategies are mainly designed to achieve the control of mother's HIV replication. Indeed, a high maternal HIV_1_ RNA plasma viral load (pVL) during the last trimester of pregnancy has been identified as the most important predictive factor for MTCT [9], [10], [11]. MTCT is also enhanced by other factors such as co-infections (chorioamniotitis, gonorrhea, herpes simplex virus 2), obstetric complications and mixed feeding [4], [10], [11], [12], [13].

Taking advantage of the PACOME trial of malaria prophylaxis which enrolled HIV-infected pregnant women, we designed an ancillary study to investigate the predictive factors associated with virologic suppression at the third trimester of pregnancy, before and after the implementation of the WHO revised guidelines for the prevention of MTCT (PMTCT) in Benin.

## Methods

### Ethics statement

The PACOME trial has been ethically approved both in France and in Benin, respectively by the “Comité consultatif de déontologie et d'éthique de l'IRD”, and by the “Comité national provisoire d'éthique pour la recherche en santé”.

### Study sites and participants

This study has been conducted in Benin, where HIV overall prevalence is around 1.2% [1] and can reach 4% in pregnant women attending antenatal care in Cotonou. Women were enrolled within the frame of the PACOME clinical trial, conducted in 5 urban hospitals in the South of Benin since December 2009 [14].

The PACOME trial was a randomized, non-blinded non-inferiority trial (NCT00970879) aiming to compare the efficacy of two strategies for malaria prevention in HIV-infected pregnant women: a daily intake of cotrimoxazole prophylaxis versus three intakes of mefloquine intermittent preventive treatment during the second and third trimesters of pregnancy. Prevalence of placental malaria was the primary endpoint. All enrolled women had to be HIV1-infected and between 16 and 28 weeks of gestation, to have signed the informed consent and to have no contra-indication to the study drugs. A total of 432 women were enrolled in the PACOME trial, from December 2009 until December 2011. For the current ancillary study, we restricted the analysis to the women who had completed the third visit or had reached 36 weeks of gestation the 15^th^ of June 2011. So, the observation period extended from seven months before to one year after the implementation of the new Beninese PMTCT recommendations (consistent with the 2009 WHO guidelines), in June 2010.

### Prevention of mother-to-child HIV transmission

Both women and infants received PMTCT in accordance with the Beninese national guidelines, in line with the WHO guidelines. Women who were already under treatment before pregnancy continued with the same ART. In other cases, ART was prescribed immediately if HIV pregnant women needed treatment for themselves (i.e. CD4 count≤350/mm^3^ and/or WHO clinical stage 3 or 4), or at different times during pregnancy according to the ongoing PMTCT guidelines: before June 2010, ART was recommended from 28 weeks of pregnancy and after June 2010 it was recommended from 14 weeks of pregnancy. The ART regimen associated two nucleosidic reverse transcriptase inhibitors (NRTIs) and one non-nucleosidic reverse transcriptase inhibitor (NNRTI), with predilection for zidovudine, lamivudine and efavirenz in the new recommendations. ART was administered to the mothers until one week after delivery or during all the breastfeeding period. According to Beninese recommendations, infants first received a single dose of nevirapine at birth and then a zidovudine-lamivudine bi-therapy for three weeks. From February 2011, new Beninese guidelines (in line with the WHO recommendations) changed to daily nevirapine for six weeks.

### Data collection

#### Socio economic and medical data

According to the PACOME trial follow-up, women were evaluated at three antenatal care (ANC) visits scheduled at least one month apart (between 16–28, 24–32 and 28–36 weeks) and at delivery. Infants' evaluations were scheduled at birth, at six weeks and four months of life, and two months after weaning in case of breastfeeding.

Socio-economic data and obstetrical history were recorded at enrolment as well as HIV disease information. On the occasion of each visit and at delivery, obstetrical, malaria and HIV-related information were also collected. Self-reported adherence to ART was collected at each visit using a standardized questionnaire, asking the woman if she had omitted her medication during the preceding month and, if so, precisely how many intakes had been omitted during the preceding week (last 7 days recall). A ratio of non-adherence was calculated as the number of omitted tablets reported to the total number of tablets to be taken during that week. At delivery, information about the type of infant's feeding and ART were collected.

#### Laboratory investigations

Before enrolment, HIV rapid diagnostic screening tests were performed first by the Determine® test, further confirmed by the Bioline® test. Women with two positive results were tested by Elisa for confirmation of HIV-1 seropositivity.

Venous samples were collected in EDTA tubes on each scheduled visit.

HIV-1 pVL was assessed twice: once at enrolment and once in late pregnancy (on the occasion of the last third trimester visit, or at delivery). Two laboratories (“Programme National de Lutte contre le Sida” in Cotonou and Clinique Louis Pasteur in Porto-Novo) assessed pVLs, both using the real-time PCR analyser m2000rt Abbott® with a 40 copies/mL detection threshold. In infants, qualitative PCRs using the same automat were planned at the age of six weeks and four months (or two months post weaning in case of breastfeeding). Absolute CD4-cell count was assessed on each scheduled visit by a Cyflow® or Facscount® cytometer, depending on the study site.

Hemoglobin level was determined as part of complete blood count (CBC) or if not available by a Hemocue® colorimeter.

Thick blood smears were performed at each scheduled visit and at additional visits in case of malaria symptoms. Blood smears were stained with Giemsa and microscopically examined for the detection of malaria parasites.

### Definitions

The primary outcome was the third trimester pVL, either performed at delivery or during the last ANC visit in the third trimester of pregnancy. In case two pVL assessments were made in the same woman, delivery pVL was selected for analysis. It was categorized into detectable (>40 copies/ml) versus undetectable, and used as a proxy to measure PMTCT efficacy.

At enrolment, pVL was dichotomized into high (>30 000 copies/ml or 4 log copies) or low. The low category gathered women with undetectable and low but detectable pVLs.

Virologic failure was determined for women already under treatment at enrolment. It was defined as the persistence of pVL>5000 copies/ml after 6 months of ART [15].

Our study focused on HIV virologic suppression at the end of pregnancy, but we also had some data about infants' follow-up available from the Beninese national program for PMTCT. When it was performed, the last result of HIV PCR determined the infant's HIV viral status, reflecting the real efficacy of PMTCT.

Women were considered to have impaired adherence if they reported omitting ART intakes at the seven-day recall, at least once during the follow-up.

Anemia was defined as a hemoglobin level below 9.5 g/dL [16].

Malaria events were defined as a positive parasitemia with or without clinical signs, or as the presence of fever resulting in the prescription of an antimalarial treatment, whatever the blood smear result.

Women were asked which ethnic group they belonged to (according to their mother's vernacular language) and were categorized into the “Fon and assimilated” ethnic group (the predominant ethnic group in Southern Benin where the study took place) or into “Other” (Mina from Western Benin and Togo, Yoruba from Eastern Benin and Nigeria, Peul and Dendi from Northern Benin…).

### Statistical analysis

First, descriptive statistics were used to specify women's characteristics. To make comparisons between periods (before and after the implementation of the revised recommendations), the Chi Square and Student tests were used respectively for categorical variables and continuous variables.

A logistic regression was then used to estimate the crude and adjusted odds ratios (ORs) for undetectable HIV pVL at the third trimester. Independent variables were first tested one by one in univariate models. When required, continuous variables were dichotomized into two categories, using either usual cut-offs from the literature or the median value if none was available. In multivariate models, forced variables were the study site, the pVL at enrolment (dichotomized with a cut-off of 30000 copies/ml) and the CD4 cell count at enrolment (with a cut-off of 350/mm^3^). Other variables showing an association with P ≤0.20 in univariate analysis were also included. A backward stepwise strategy was used to select the best restricted model in which interactions were tested. The conservative threshold was P<0.05. We finally performed sensitivity analyses, considering women with missing third trimester pVLs in the « detectable » and « undetectable » groups successively. The statistical analysis was performed using STATA® (version 11).

## Results

**Figure 1:**
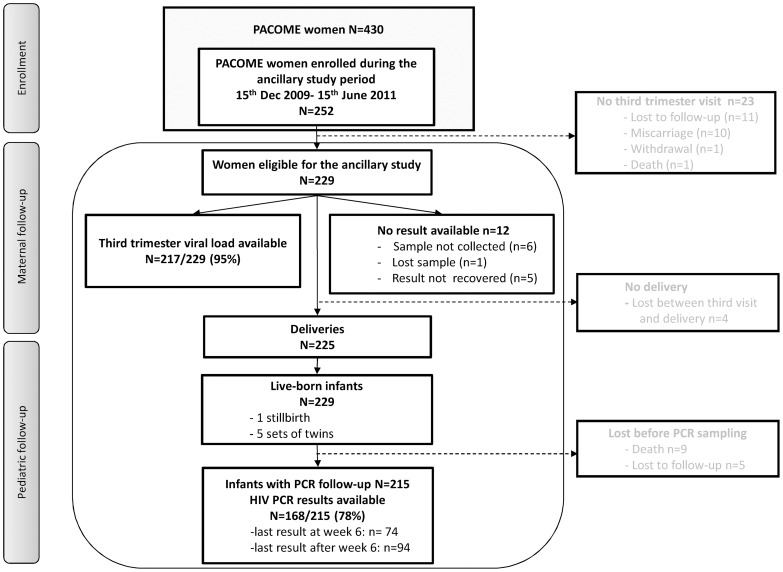
Study flow-chart. Women were eligible for the ancillary study if they had been enrolled during the study period and had their third trimester visit performed.

A total of 252 pregnant women were enrolled during the study period (**Figure 1**), among whom 23 were lost before the third trimester visit, when viral load was measured: 10 experienced miscarriages, 11 were lost to follow-up, one died of HIV-related wasting syndrome, one refused to continue participation.

Among the eligible sample of 229 women with a visit performed at the end of pregnancy, the HIV pVL results were not available for 12 women (6 uncollected blood samples, one sample lost, 5 results not retrieved). Finally, a total of 217 third trimester pVLs were available for analysis. Characteristics of the 217 women are described in **Table 1**. They were generally comparable to the 12 women excluded (data not shown). However, women with missing pVLs were more likely to have been enrolled at the beginning of the study and to come from the Porto-Novo site, the most distant from the PACOME laboratory. In our population, most of the women (72%) were at an advanced stage of the HIV disease and needed ART for themselves. The mean CD4 count on inclusion was 361/mm^3^. The pVL decreased from enrolment (mean, 2.9 log) until the third trimester of pregnancy (mean, 1.5 log), when 71% of women had an undetectable pVL. The median gestational age at this last pVL determination was 35 weeks of pregnancy, i.e. one month prior to delivery.

**Table 1:** Women's characteristics (N = 217).

Category	Subcategory	n or *mean*	% or *SD*
Characteristics at enrolment			
Study Site	CNHU	85	39
	HIA Camp Guezo	40	18
	Clinique Louis Pasteur	10	5
	Hôpital de zone Suru Lere	62	29
	Hôpital HOMEL	20	9
Age (years)		*29.3*	*4.9*
Ethnic group	Fon and assimilated^1^	99	46
	Others	118	54
Occupation	None or informal job	192	88
	Regular job	25	12
Ever attended school (N = 215)		154	72
Higher socio-economic status^2^ (N = 215)		97	55
Marital status	Single	17	8
	Married/with partner	200	92
Rank of gestation	Primi	24	11
	Secondi	46	21
	Multi	147	68
History of miscarriage or stillbirth		114	53
Gestational age at enrolment (weeks)		*21.4*	*3.8*
Weight at enrolment (Kg)		*62.9*	*11.6*
Number of ANC visits before enrolment		*1.7*	*1.1*
Time since HIV diagnosis^3^ (months) (N = 208)		*15.4*	*1.4*–*41.0*
WHO HIV stage 1–2		182	84
CD4 cell count (/mm^3^)		*362*	*198*
ART initiated before enrolment		122	66
Median timing since ART initiation (months) (N = 121)		*16.1*	*1.9*–*41.5*
HIV viral load at enrolment (log/ml) (N = 208)		*2.9*	*1.8*
	In women not treated (N = 92)	*4.1*	*1.2*
	In women already treated (N = 116)	*1.9*	*1.6*
Virologic failure at enrolment^4^		13	6
Undetectable at enrolment		65	30
Father's HIV serology	Unknown	107	49
	Negative	59	27
	Positive	51	24
Characteristics during follow-up			
Total number of ANC visits		*6*	1.7
Third trimester HIV viral load (log copies/ml)		*1.5*	*1.2*
Undetectable third trimester HIV viral load (<40 copies/ml)		155	71
Third trimester CD4 cell count (/mm^3^) (N = 212)		*439*	*278*
Anemia (<9.5g/dl) during pregnancy		83	38
Gestational age at delivery (weeks of pregnancy) (N = 213)		*38.4*	*1.7*
Premature delivery		18	8
Malaria during pregnancy		31	14
Gestational age at malaria diagnosis^5^ (weeks of pregnancy) (N = 31)		*18.7*	*9.0*

SD, standard deviation; IQR, interquartile range; ART, antiretroviral therapy; ANC, antenatal care.

1 The Fon ethnic group is the predominant ethnic group in South of Benin where the study took place.

2 Defined by four or more of the pre-specified household assets: toilets, electricity, a refrigerator, television, motorbike, car.

3 Median value and interquartile range are presented for the time since HIV diagnosis; 43% of enrolled women had been diagnosed during the ongoing pregnancy.

4 Virologic failure defined as the persistence of pVL>5000 copies/ml after 6 months of ART.

5 If more than one malaria event (n = 6), the gestational age at the first event was considered.

**Table 2** describes the PMTCT measures followed by women and infants according to the period at enrolment (before or after the change in recommendations). Among women who had started the treatment during pregnancy (62% overall), ART was initiated on average two weeks earlier after the change in recommendations (21 weeks after versus 23 weeks before, P = 0.046). An earlier initiation of ART was precisely the objective of the new recommendations. Similarly, the women were more likely to receive efavirenz than nevirapine-based ART after the change, which was also concordant with the new guidelines. Impaired adherence defined by omitted intakes at least once during follow-up concerned 28 women (13%). Two of them were 100% non-adherent during the whole follow-up, though treatment had been prescribed. For the remaining 26, the percentage of non-adherence ranged from 5% to 100% of omitted intakes. The main reasons advocated for omitting ART intakes were “simple omission”, followed by stock shortage.

**Table 2:** characteristics of the PMTCT measures according to the period at enrolment (before or after the implementation of the revised guidelines in Benin).

		Total		Enrolled before revised guidelines		Enrolled after revised guidelines		P
Category	Subcategory	N or *mean*	% or *SD*	N or *mean*	% or *SD*	N or *mean*	% or *SD*	
Ante partum		N = 217		N = 72		N = 145		
HIV diagnosis (N = 217)	Before pregnancy	123	57	40	56	83	57	0.813
	During pregnancy	94	43	32	44	62	43	
Gestational age at diagnosis (weeks of pregnancy) (N = 94)		*15.4*	*5.9*	16.4	5.4	15.0	6.2	0.282
HIV viral load at enrolment (log/ml) (N = 208)		*2.9*	*1.8*	3.2	2.0	2.7	1.7	0.078
Indication for ART	For PMTCT alone	61	28	14	19	47	32	0.045
	For themselves	156	72	58	81	98	68	
Timing for ART initiation	Before pregnancy	81	38	26	37	55	38	0.882
	During pregnancy	136	62	46	63	90	62	
Gestational age at ART initiation (weeks of pregnancy) (N = 135)^2^		*21.6*	*6.5*	*23.2*	*6.9*	*20.8*	*6.1*	0.046
	≤15	22	10	4	6	18	12	0.029
	16–27	89	41	28	39	61	42	
	>28 or no treatment	24	11	13	18	11	8	
ART regimen	With nevirapine	96	44	44	61	52	36	<0.0001
	With efavirenz	114	53	22	31	92	63	<0.0001
	With PI	4	2	3	4	1	1	0.07
	No ART	2	1	2	3	0	0	–
	Unknown	1	<0.5	1	1	0	0	–
Impaired adherence to ART		28	13	18	25	10	7	<0.0001
Intra partum		N = 212		N = 75		N = 137		
Caesarean section (N = 212)^1^		64	30	21	28	43	31	0.642
Post-Partum		N = 222		N = 81		N = 141		
Infant feeding (N = 220)	Breast milk 3	133	60	47	59	86	61	0.696
	Formula milk	87	40	33	41	54	39	
	Mixed	0	0					
ART in the infant (N = 222)	Single dose NVP and 3 weeks AZT/3TC	194	87	77	95	117	83	<0.0001
	NVP 6 weeks	21	10	0	0	21	15	
	Single dose NVP alone	1	<0.5	1	1	0	0	
	No ART	7	3	3	4	3	2	

SD, standard deviation; IQR, interquartile range; ART, antiretroviral therapy, PMTCT, prevention of mother-to-child transmission; ANC, antenatal care. P values obtained by chi-square or student test.

1 Overall, caesarean section was indicated for PMTCT in 22% of cases, other cases had obstetrical indications.

2 calculated for initiation during pregnancy.

3 The median breastfeeding duration was 6 months (maximum one year).

After delivery, 60% of the women chose breastfeeding. Three percent of the infants (n = 6) failed to receive any ART, because of missed appointment or delivery in a non-qualified site.

The univariate and multivariate logistic regressions modeling the risk of having an undetectable third trimester pVL are shown in **Table 3** and **Table 4**, respectively.

**Table 3:** Factors associated with an undetectable HIV_1_ RNA plasma load at the end of pregnancy; univariate logistic regression (N = 217).

Category	Subcategory	n/N (%) or *mean* 1	OR [95%CI]	P
Study site				0.432
Ethnic group	Fon and assimilated^1^	64/99 (65)	1	0.044
	Others	91/118 (77)	1.85 [1.02–3.33]	
Age (years)^2^		*29.8 vs 28.0*	1.09 [1.01–1.15]	0.018
Rank of gestation	Multi	113/147 (77)	1	0.011
	Primi/secondi	42/70 (60)	0.45 [0.24–0.83]	
Occupation	None or informal job	142/192 (74)	1	0.026
	Regular job	13/25 (52)	0.38 [0.16–0.89]	
Ever attended school	No	39/61 (64)	1	0.128
	Yes	116/154 (74)	1.64 [0.87–3.13]	
WHO HIV stage	1–2	126/182 (69)	1	0.108
	3–4	29/35 (83)	2.13 [0.85–5.56]	
CD4 cell count (/mm^3^)	≤350	72/115 (63)	1	0.003
	>350	83/102 (81)	2.61 [1.40–4.88]	
HIV_1_ RNA load (copies/ml) (N = 208)	≤30000	125/154 (81)	1	<0.0001
	>30000	21/54 (39)	0.15 [0.07–0.29]	
ART initiated before enrolment	No	61/95 (64)	1	0.039
	Yes	94/122 (77)	1.89 [1.03–3.45]	
Virologic failure at enrolment	No	144/195 (74)	1	<0.0001
	Yes	2/13 (15)	0.06 [0.01–0.30]	
Knowledge of the father's serology	No	71/107 (66)	1	0.104
	Yes	84/110 (76)	1.64 [0.90–2.94]	
Enrolled while new recommendations were implemented	No	42/72 (58)	1	0.003
	Yes	113/145 (73)	2.50 [1.37–4.55]	
History of miscarriage or stillbirth	No	69/103 (67)	1	0.170
	Yes	86/114 (75)	1.52 [0.84–2.70]	
Gestational age at enrolment (weeks)	≤21	89/117 (76)	1	0.103
	>21	66/100 (66)	0.61 [0.34–1.10]	
Weight at enrolment (Kg) 2		*64.3 vs 59.4*	1.04 [1.01–1.08]	0.006
Indication for ART	For PMTCT alone	48/61 (79)	1	0.141
	For themselves	107/156 (69)	0.59 [0.29–1.19]	
Timing of ART initiation (weeks of pregnancy)	≤28	145/193 (75)	1	0.001
	>28	10/24 (42)	0.24 [0.10–0.57]	
Time elapsed between ART initiation and viral load measurement (weeks)	≤8	12/29 (41)	1	<0.0001
	>8	143/188 (76)	4.50 [2.00–10.13]	
Impaired adherence to ART	No	141/189 (75)	1	0.009
	Yes	14/28 (50)	0.34 [0.15–0.76]	
Number of ANC visits	≤6	95/145 (65)	1	0.007
	>6	60/72 (83)	2.63 [1.30–5.26]	
Anemia (<9.5g/dl) during pregnancy	No	105/134 (78)	1	0.005
	Yes	50/83 (60)	0.42 [0.23–0.76]	
Malaria during pregnancy	No	135/186 (73)	1	0.046
	Before 18 weeks^3^	14/17 (83)	1.76 [0.49–6.39]	
	After 18 weeks	6/14 (43)	0.28 [0.09–0.86]	

OR, odds ratio; CI, confidence interval; ART, antiretroviral therapy, PMTCT, prevention of mother-to-child transmission; ANC, antenatal care. The P values presented were computed with the Wald test.

1 The Fon ethnic group is the predominant ethnic group in South of Benin where the study took place.

2 For categories, the proportion of women with undetectable HIV1 RNA load is presented. For continuous variables, the mean in the undetectable group versus detectable group is presented. For continuous variables, means in the undetectable group versus detectable group are presented.

3 For the 6 patients who presented with two consecutive malaria episodes, the gestational age at the first one was considered.

**Table 4:** Factors associated with an undetectable HIV_1_ RNA plasma load at the end of pregnancy; multivariate logistic regression (N = 208).

Category	Subcategory	AOR [95%CI]	P
Study site^1^			0.778
Ethnic group	Fon and assimilated^2^	1	0.004
	Others	3.61 [1.50–8.71]	
Rank of gestation	Multi	1	0.088
	Primi/Secondi	0.45 [0.18–1.13]	
Occupation	None or informal job	1	0.003
	Regular job	0.17 [0.05–0.56]	
CD4 cell count (/mm^3^) 1	≤350	1	0.086
	≤350	2.17 [0.90–5.27]	
HIV_1_ RNA load at enrolment (copies/mL)	≤30 000	1	<0.0001
	>30000	0.19 [0.08–0.46]	
Virologic failure at enrolment	No	1	<0.0001
	Yes	0.03 [0.004–0.21]	
Weight at enrolment (Kg)		1.05 [1.01–1.10]	0.020
Time elapsed between ART initiation and viral load measurement (weeks)	≤8	1	0.002
	>8	5.52 [1.84–16.61]	
Impaired adherence	No	1	0.067
	Yes	0.35 [0.11–1.08]	
Number of ANC visits	≤6	1	0.014
	>6	3.55 [1.30–9.72]	

AOR, adjusted odds ratio; CI, confidence interval; ART, antiretroviral therapy, PMTCT, prevention of mother-to-child transmission; ANC, antenatal care. The P values presented were computed with the Wald test. Of the 217 women with a third pregnancy HIV viral load determined, 9 were not included in the multivariate analysis because the HIV viral load at enrolment was missing.

1 Adjustment covariates forced in the multivariate models (for study site, 4 dummy variables).

2 The Fon ethnic group is the predominant ethnic group in South of Benin where the study took place.

In univariate analysis (**Table 3**), factors increasing the probability to obtain virologic suppression were: older age, higher weight, higher CD4 cell count at enrolment and longer course of ART. Women enrolled after the new recommendations were implemented were also more likely to achieve virologic suppression (odds ratio (OR)  = 2.50, 95% confidence interval (CI)  = 1.37–4.55). Factors negatively related were the “Fon and assimilated” ethnic group, having a regular job, first or second pregnancy (60% of virologic suppression in primi- and secondigravid women versus 77% in multigravid women, P = 0.01), a high pVL at enrolment, virologic failure at enrolment, and an impaired adherence to ART. Anemia during follow-up was also inversely associated with an undetectable pVL, as well as the occurrence of a malaria episode after 18 weeks of pregnancy (OR  = 0.28, 95% CI  = 0.09–0.86). No differential effect was observed according to the type of ART regimen in this population almost exclusively treated with NNRTIs.

In multivariate analysis (**Table 4**), models were forced on the study center, the CD4 cell count and the pVL at enrolment. Among baseline characteristics which remained independently associated, higher weight and marginally higher CD4 cell-count increased the probability of virologic suppression at the end of pregnancy. First or second pregnancy, Fon ethnic group, regular job, higher baseline pVL and virologic failure at enrolment decreased the probability of having an undetectable pVL, by more than thirty fold in the last case (OR  = 0.03, P<0.0001). Of interest, some follow-up characteristics were very strongly associated with undetectable pVL, such as early initiation of ART (OR  = 5.52, 95% CI  =  [1.84–16.61] for ART started more than 8 weeks before the measurement of pVL) and high antenatal care attendance (OR  = 3.55, 95% CI  =  [1.30–9.72] if the women had attended more than 6 antenatal visits during pregnancy). Impaired adherence to ART during pregnancy had a borderline negative effect (OR  = 0.35, 95% CI  = 0.11–1.08, P = 0.067). The effect of the period at enrolment (before or after the change in recommendations) did not remain significant after adjusting for the duration of ART. The effects of anemia and malaria also vanished in multivariate analyses. A sensitivity analysis took into account the 12 women whose third trimester pVL was missing, considering them successively in the detectable then in the undetectable group. These analyses showed consistent results (see **Table S1**).

Finally, HIV status could be determined by PCR in 168/215 (78%) of the infants born to the women in our cohort (figure 1). These PCRs were performed by the Beninese National Program and did not rely on the PACOME trial. PCR determinations were often not available later than 6 weeks after birth, so positive results accounted more for pre and intrapartum than for postpartum HIV transmission in these predominantly breastfed infants. Three infants had a positive PCR result, so the MTCT rate accounting for early contamination was 1.9% (95% CI 0.3%–5.4%). The mothers of all 3 contaminated infants had a pVL over 30 000 copies/mL at enrolment and were still detectable at the end of pregnancy. Details about the three cases of MTCT are given in **Table S2**.

## Discussion

This study, aiming to identify the factors associated with an undetectable pVL at the third trimester of pregnancy, is the first to report results about a large cohort of HIV-infected pregnant women in Benin. In this cohort, 71% of the women achieved virologic suppression before delivery, in keeping with the observations made in other cohorts of women receiving triple ART in industrialized countries (USA, 68% [17], Europe, 73% [18] and 77% [19]) and South Africa (Botswana, 94% [20]). In infants born to the mothers of the present study, the PCR-MTCT rate accounting for pre and intra-partum contamination was around 2%, concordant with other studies [20], [21], [22].

An important finding of this study is that we confirmed the importance of early initiation of ART in pregnancy. The longer the ART was taken, the higher was the probability to achieve virologic suppression at the end of pregnancy. This result corroborates previous studies [18], [21], [23] and the WHO 2009 recommendations, which advocated for ART initiation at 14 weeks of pregnancy. A recent study demonstrated that extended use of ART during pregnancy correlated with significant reduction of MTCT [24]. Our data suggest it could be mediated by virologic suppression. Updated 2012 recommendations indicate that pregnant women diagnosed HIV-positive should be put on ART immediately [25], whether they need treatment for themselves or not, and without discontinuation after delivery. We showed that the probability to have an undetectable pVL was more than 4-fold increased if the treatment lasted for 8 weeks or more. Such duration could be achieved only if ART was initiated before 28 weeks of gestational age (i.e. the third trimester of pregnancy). The change in Beninese recommendations during the study period resulted in an earlier initiation of ART, illustrating good adherence to the new guidelines. As the change in PMTCT recommendations occurred while the trial was going on, 72 women were enrolled before the new guidelines were implemented and 145 were enrolled afterwards, ensuring a sufficient power (83%) to detect an increase in virologic suppression rate from 60% to 80% between the two periods.

Another important finding is the crucial role of antenatal care attendance, which was particularly high in our study (median of 6 antenatal visits recorded) with a direct impact on the control of the mother's pVL. This study was performed in the framework of a clinical trial, thus restricting extrapolation to routine practice. However, previous observational studies set in Benin showed that most pregnant women spontaneously attended at least 3 antenatal visits and sometimes up to 8, from the beginning of the second trimester of pregnancy [26], [27]. Such high antenatal care coverage and attendance represent a real advantage as compared to other African settings, and might be one of the reasons why the results obtained in this study were particularly good.

The severity of HIV disease at enrolment such as baseline CD4 cell count and pVL were determinant factors that corroborated the results of previous studies [17], [23], [28]. A recent study showed that a baseline pVL over 10 000 copies/ml strongly decreased the probability to reach undetectability at delivery, and pointed out that ART had to be initiated before 20 weeks of pregnancy [19]. Women in their first or second pregnancy were also at higher risk in our cohort, as they were more likely to have been diagnosed HIV-infected during the ongoing pregnancy and to be treatment-naïve.

The negative effect of a high pVL at enrolment was more marked if women had already started ART and had virologic failure [15]. We believe that virologic failure was subsequent to an acquired resistance to treatment (even though resistance genotyping was not available here). Only two of the 13 women with virologic failure switched to a second line of ART containing protease inhibitors, one of them showing impaired adherence during pregnancy. Expectedly [29], [30], the women in our cohort who reported impaired adherence to ART also had a lower probability to achieve virologic suppression.

Regarding malaria, it has been found to increase pVL in previous studies [31], [32], [33]. Our data showed a similar effect of malaria in univariate analysis only. Women who presented with malaria after 18 weeks of pregnancy (i.e., after enrolment) also reported more frequently an impaired adherence to ART. ART adherence was correlated with cotrimoxazole adherence during follow-up (data not shown), and possibly also to other malaria prevention strategies like the use of insecticide-treated bed nets. Multivariate analysis did not confirm the effect of malaria on pVL and we think the best explanation is the confounding induced by impaired adherence to both ART and cotrimoxazole, rather than a direct effect of parasites on maternal immunity and HIV replication.

More unexpected results appeared, such as the negative effect of having a regular job. Women in the formal sector had a higher socio-economic status, but they were also younger and more likely to be on their first or second pregnancy, which factors were negative.

A particularly surprising result found in univariate analysis and confirmed in multivariate analysis was the effect of the ethnic group on the third trimester pVL. Women from the “Fon and assimilated” ethnic group, i.e. the predominant ethnic group in this part of Benin, were less likely to reach virologic suppression. These women native from the study area, speaking the local language, and usually living with their family, were expected to better achieve PMTCT goals. This finding might be attributed to socio-anthropological as well as genetic particularities in this ethnic group, beyond the scope of our study. It needs to be confirmed by other studies.

Missing determinations of some HIV viral loads in mothers and PCRs in infants may constitute a weakness of the study. Twelve women had no HIV viral load performed at the end of pregnancy. We do not think these missing data could have induced a bias, since most of the baseline characteristics did not differ between excluded and included women. Notably, the time elapsed since HIV diagnosis, the baseline HIV viral load and the time since ART initiation were similar in both groups. Sensitivity analysis also showed consistent results. In the infants born from the cohort, some HIV PCRs were missing (22%). Overall, missing values were mostly due to logistic dysfunctions in the National PMTCT Program laboratory. Regarding mothers belonging to the PACOME trial, all procedures were carefully scheduled and secured during follow-up, allowing us to retrieve most of the results even if they were available after delivery. Unfortunately, the same quality of follow-up did not apply to the infants who were treated according to the national standards of care. Routine virologic monitoring is indeed a challenge in many resource-poor settings [34]. Missing values in infants thus limited our possible interpretation of the impact of undetectable maternal pVL on the rate of mother-to-child transmission of the virus.

To conclude, two major results can be drawn from this study. First, for women already on ART before pregnancy, it appears crucial to recommend a systematic determination of pVL at the beginning of pregnancy, in order to detect virologic failure and eventually switch to a second line ART. Second, for women not already diagnosed HIV-infected, a key component of PMTCT is to propose HIV screening early in pregnancy, in order to initiate ART promptly. ART initiation before the third trimester of pregnancy is a good predictor of virologic suppression before delivery and ensures to minimize the risk of pre and intra-partum MTCT. In Benin, a good implementation of the new recommendations supporting early initiation combined to a high antenatal care attendance led to fine results. The high coverage and high attendance of antenatal care are indeed critical for the implementation of effective public health policies dedicated to the virtual elimination of MTCT.

## Supporting Information

Table S1 **Sensitivity analysis of the factors associated with an undetectable HIV_1_ RNA plasma load at the end of pregnancy taking into account missing values; multivariate logistic regression.**
(DOC)

Table S2 **Description of the MTCT cases (positive HIV PCR at 6 weeks of life).**
(DOC)
