# Supplementary material for: Predictive Factors of Plasma HIV Suppression during Pregnancy: A Prospective Cohort Study in Benin
Source: PLoS One. 2013 Mar 15;8(3):e59446. doi: 10.1371/journal.pone.0059446 (PMC3598754; doi:10.1371/journal.pone.0059446)
Supplement: Table S1 — Sensitivity analysis of the factors associated with an undetectable HIV1 RNA plasma load at the end of pregnancy taking into account missing values; multivariate logistic regression. (DOC) [file pone.0059446.s001.doc]

**Table S1. Sensitivity analysis of the factors associated with an undetectable HIV_1_ RNA plasma load at the end of pregnancy taking into account missing values; multivariate logistic regression.**

|  | **Pessimistic scenario** | | **Optimistic scenario** | |
| --- | --- | --- | --- | --- |
|  | AOR [95%CI] | P | AOR [95%CI] | P |
| Study site**^1^** |  | 0.330 |  | 0.617 |
| Ethnic group  Fon and assimilated  Others | 1  2.87 [1.32-6.25] | 0.008 | 1  2.74 [1.19-6.33] | 0.018 |
| Rank of gestation  Multigravid  Primi/Secondi |  |  | 1  0.39 [0.16-0.92] | 0.032 |
| Ever attended school  No  Yes |  |  | 1  2.70 [1.07-6.79] | 0.035 |
| Occupation  None or informal job  Regular job | 1  0.17 [0.06-0.51] | 0.001 | 1  0.12 [0.04-0.42] | 0.001 |
| CD4 cell count (/mm3)**^1^**  ≤ 350  > 350 | 1  1.34 [0.61-2.96] | 0.467 | 1  2.10 [0.89-4.93] | 0.089 |
| HIV_1_ RNA load at enrolment (copies/mL)^1^  ≤30 000  >30000 | 1  0.21 [0.09-0.5] | <0.001 | 1  0.26 [0.11-0.62] | 0.002 |
| Virologic failure at enrolment  No  Yes | 1  0.04 [0.01-0.27] | 0.001 | 1  0.04 [0.01-0.25] | <0.001 |
| Weight at enrolment (Kg) | 1.05 [1.01-1.08] | 0.016 |  |  |
| Time elapsed between ART initiation and viral load measurement (weeks)  ≤ 8  > 8 | 1  4.59 [1.63-12.96] | 0.004 | 1  6.48 [2.18-19.25] | 0.001 |
| Impaired adherence  No  Yes | 1  0.29 [0.11-0.78] | 0.014 |  |  |
| Number of ANC visits  ≤ 6  >6 | 1  3.48 [1.39-8.68] | 0.008 | 1  3.78 [1.41-10.1] | 0.008 |
| Anemia during pregnancy  No  Yes |  |  | 1  0.36 [0.15-0.83] | 0.016 |

AOR, adjusted odds ratio; CI, confidence interval; ART, antiretroviral therapy, ANC, antenatal care. In the pessimistic scenario, women with missing values for the third trimester HIV viral load were considered in the detectable group, whereas in the optimistic scenario thy were considered in the undetectable group (so the proportion of virologic suppression ranged from 68% to 73%). The P values presented were computed with the Wald test. Of the 229 eligible women, 11 were not included in the multivariate analysis because the HIV viral load at enrolment was missing and 1 because the data about adherence were missing.

**^1^** Adjustment covariates forced in the multivariate models (for study site, 4 dummy variables).
