# Supplementary material for: Predictive Factors of Plasma HIV Suppression during Pregnancy: A Prospective Cohort Study in Benin
Source: PLoS One. 2013 Mar 15;8(3):e59446. doi: 10.1371/journal.pone.0059446 (PMC3598754; doi:10.1371/journal.pone.0059446)
Supplement: Table S2 — Description of the MTCT cases (positive HIV PCR at 6 weeks of life). (DOC) [file pone.0059446.s002.doc]

**Table S2. Description of the MTCT cases (positive HIV PCR at 6 weeks of life).**

|  | Rank of gestation | CD4 cell count at enrolment  (/mm3) | HIV RNA viral load at enrolment  (copies/ml) | Maternal ART regimen | Timing of  ART  Initiation  (weeks) | Time elapsed between ART initiation and viral load (weeks) | Impaired adherence | HIV RNA viral load at the third trimester of pregnancy  (copies/ml) | Delivery mode | Infant ART regimen for PMTCT | Feeding | Infant outcome |
| --- | --- | --- | --- | --- | --- | --- | --- | --- | --- | --- | --- | --- |
| 1 | Primi | 121 | 1 288 250 | 3TC  AZT  EFV | 19 | 21 | No | 1 187 937 | Caesarean section | sdNVP AZT  3TC | Formula | Still alive at 4 months |
| 2^1^ | Primi | 220 | 187 324 | 3TC  D4T  NVP | 22 | 14 | Yes^2^ | 188 | Caesarean section | sdNVP AZT  3TC | Maternal | Still alive at 11 months |
| 3 | Secondi | 205 | 46 117 | 3TC  D4T  NVP | Before pregnancy | 37 | No | 33 148 | Natural^3^ | sdNVP AZT  3TC | Formula | Died at 3 months |

MTCT, mother-to-child transmission; ART, antiretroviral therapy;3TC, lamivudine; AZT, zidovudine; D4T, stavudine; EFV, efavirenz; NVP, nevirapine; sdNVP, single dose nevirapine.

^1^ Case 2 occurred while new recommendations were implemented

^2^ The woman reported an adherence of 93% at the second visit (100% at 3 other visits)

^3^ Delivery occurred in the study centre and was secured with hygiene precautions
